# Reduced expression of *BIRC2* and *BIRC3* associated with longer survival in pediatric high-grade gliomas

**DOI:** 10.1038/s41598-026-35887-7

**Published:** 2026-01-30

**Authors:** Alicja Petniak, Paulina Gil-Kulik, Julia Zarychta, Adrian Kowalczyk, Joanna Trubicka, Marta Perek-Polnik, Cezary Grochowski, Ryszard Maciejewski, Wiesława Grajkowska, Janusz Kocki

**Affiliations:** 1 Department of Clinical Genetics, Medical University of Lublin, 11 Radziwillowska Str, Lublin, 20-080 Poland; 2 Student Scientific Society of Clinical Genetics, Medical University of Lublin, 11 Radziwillowska Str, Lublin, 20-080 Poland; 3 Doctoral School, Medical University of Lublin, Lublin, Poland; 4 Department of Pathology, The Children’s Memorial Health Institute, Warsaw, Poland; 5 Department of Oncology, The Children’s Memorial Health Institute, Warsaw, Poland; 6 Institute of Health Sciences, The John Paul II Catholic University of Lublin, Konstantynów 1 str, Lublin, Poland

**Keywords:** Pediatric high grade glioma, IAP family, BIRC, p53, Olig2, Ki67, GFAP, Synaptophysin, Cancer, Cell biology, Genetics, Molecular biology, Oncology

## Abstract

Inhibitors of apoptosis proteins (IAPs), coded by *BIRC* genes, are cellular checkpoints that can regulate and inhibit pro-apoptotic caspase signaling. Overexpression of *BIRC* genes has been associated with cancer progression, multidrug resistance, poor prognosis, and shorter survival in several types of cancer. Using quantitative real-time polymerase chain reaction, we examined the expression of IAP family genes and their regulators: *NAIP*, *BIRC2*, *BIRC3*, *XIAP*, *BIRC5*, *BIRC6*, *BIRC7*, *CASP3*, *CASP9*, *DIABLO* and *XAF1*. We also evaluated the impact of clinical parameters (programmed death receptor 1 [PD1] expression, oligodendrocyte transcription factor 2 [Olig2] expression, Ki-67 antigen expression, tumor protein p53 expression in tumor cells, patient survival time, and progression-free survival) on gene expression levels. The expression of *BIRC3* (*p* = 0.049), *NAIP* (*p* = 0.008), and *XAF1* (*p* = 0.032) was significantly higher in tumors negative for Ki67, whereas the remaining genes showed no significant correlation with Ki67 expression. In contrast, *BIRC2* (*r*=-0.478 *p* < 0.05) and *BIRC3* (*r*=-0.536 *p* < 0.05) expression levels were negatively correlated with overall survival. A similar negative association was observed between progression-free survival and the expression of *BIRC2* (*r*=–0.481, *p* < 0.05) and *BIRC3* (*r*=-0.540, *p* < 0.05). To our knowledge, this is the first study to comprehensively assess the relationship between the expression of IAP family genes and their regulators in a homogeneous group of patients diagnosed with pediatric high-grade gliomas (pHGGs). Our findings provide new insights into molecular mechanisms involved in the pathogenesis of pHGGs, however, these preliminary results require confirmation in larger and more detailed studies.

**Supplementary Information:**

The online version contains supplementary material available at 10.1038/s41598-026-35887-7.

## Introduction

Central nervous system (CNS) tumors are the most common solid tumors in childrenand are responsible for the largest number of cancer-related deaths in pediatric population. Approximately 50% of all solid CNS tumors are gliomas^1^. According to the World Health Organization (WHO) classification, pediatric gliomas are divided into histological grades I-II (low-grade gliomas pLGG) and grades III-IV (high-grade gliomas, pHGG)^2^.

pHGGs are less common and account for 8–12% of all CNS tumors in pediatric patients but are responsible for about 40% of deaths due to CNS tumors^1^. The standard treatment of choice is surgical resection, followed by radiotherapy and adjuvant chemotherapy. However, despite intensive treatment, the prognosis in these patients remains poor (long-term survival rate below 10%)^2,3^.

In addition, intensive combined therapy is not always possible, for example, due to the patient’s very young age in cases where adjuvant radiation is contraindicated or when the tumor’s primary location and infiltrative nature limit the possibility of surgical resection^2,4–10^. In such cases, only palliative treatment can be offered, as chemotherapy currently usedin therapeutic protocols remains insufficientlyeffective in pHGG^5^.

Despite comparable morphological features of pHGG and high-grade gliomas occurring in adults (aHGG), significant genetic and molecular differences between pHGG and aHGG have been identified over the last decade^6,8,9,11^. A better understanding of tumor biology has partly explained the unsatisfactory outcomes of therapeutic strategies used to treat pHGG, as these were developed based on results from studies of aHGG^12^.

One of the common molecular mechanisms whose alterations may contribute to carcinogenesis is the dysregulation of programmed cell death (apoptosis). IAPs (inhibitors of apoptosis proteins) are cellular checkpoints that regulate and inhibit pro-apoptotic caspase signaling. Resistance to apoptotic stimuli is considered one of the main causes of cancer development, and the overexpression of IAPs is one of the mechanisms by which cancer cells acquire resistance to pro-apoptotic signals^13^. Eight mammalian IAP proteins have been identified, each containing a characteristic baculoviral IAP repeat (BIR) domain encoded by the *BIRC1–BIRC8* genes^14–16^.

The overexpression of *BIRC* genes has been associated with cancer progression, multidrug resistance, poor prognosis and shorter survival in several cancers, including pHGG, as well as in breast, colorectal, pancreatic, and lung cancers, neuroblastoma, glioblastoma (GB), melanoma, and others^14,15,17–23^.

Proteins belonging to the IAP family contain one to three characteristic baculoviral IAP repeat (BIR) domains at the N-terminus, which are important for their anti-apoptotic activity^24–26^. Additionally, they include other relevant domains, such as a caspase recruitment domain, a C-terminal RING zinc-finger domain, and a C-terminal ubiquitin-conjugating domain^27–29^.

IAPs are able to control cell survival and death through both receptor and mitochondrial pathways by inhibiting the activity of three caspases: caspase-3, caspase-7, and caspase-9^16^. Another mechanism involves the association of the BIR domain with the active site of caspases. In addition to their direct inhibitory effect on caspases, IAPs also play an important role in regulating nuclear factor kappa-light-chain enhancer of activated B cells (NF-κB) signaling pathway^30^.

A brief description of the IAP family proteins is presented in Table 1.

**Table 1:** Characteristics of the IAP family proteins.

IAP family member	Encoding gene	Gene location	Protein function	References
NAIP	*BIRC1*	5q13	Counteracting the autoactivation of pro-caspase-9 and consequently, the activation of pro-caspase-3 by caspase-9.	^31–34^
cIAP1	*BIRC2*	11q22.2	Binds to tumor necrosis factor receptor-associated factors; acts as an E3 ubiquitin ligase.	^35–37^
cIAP2	*BIRC3*	11q22.2	Binds to tumor necrosis factor receptor-associated factors; acts as an E3 ubiquitin ligases	^35–37^
XIAP	*BIRC4*	Xq25	Directly binds and inhibitis of caspase-3 activity; inhibits caspase-7 activity via the linker between BIR1 and BIR2 domains; specifically inhibits caspase-9 activity via the BIR3 domain.	^38–41^
Survivin	*BIRC5*	17q25	Binds and inhibits of caspases-3, -7 and − 9	^42,43^
BRUCE / Apollon	*BIRC6*	2p22.3	Inhibits caspases-3, -7 and − 9; promotes ubiquitination and proteasomal degradation of Smac protein	^44,45^
Livin	*BIRC7*	20q13.33	Inhibits caspases-3, -7 and − 9; inhibitsSmac/DIABLO protein	^24,46,47^
ILP-2	*BIRC8*	19q13.3-13.4	Inhibits BAX-induced apoptosis	^48,49^

IAP - inhibitor of apoptosis proteins; NAIP – neuronal apoptosis inhibitors protein; cIAP1 – cellular inhibitor of apoptosis protein-1; cIAP-2 – cellular inhibitor of apoptosis protein-2; XIAP – X-linked inhibitor of apoptosis; ILP-2 – inhibitor of apoptosis protein-related-like protein 2; BIR1/2/3 – baculoviral IAP repeat 1/2/3; Smac – second mitochondria-derived activator of caspase; DIABLO – direct inhibitor of apoptosis-binding protein with LOw pI; BAX - Bcl-2 Associated X-protein.

Many factors play an important role in the pathogenesis of gliomas, including programmed death receptor 1 (PD-1), oligodendrocyte transcription factor 2 (Olig2), antigen Ki-67 (Ki67), glial fibrillary acidic protein (GFAP), tumor protein p53 (p53) and synaptophysin. PD-1 is a common immunosuppressive checkpoint and is expressed in cytotoxic T lymphocytes, macrophages, B cells, dendritic cells, monocytes and natural killer (NK) cells in response antigen exposure^50^. Programmed cell death ligand 1 (PD-L1)/PD-1 axis over-signaling promotes T cell apoptosis and the induction of regulatory T cells (Treg) proliferation in GB microenvironments^51,52^. Olig2 is a basic-helix-loop-helix transcription factor identified as a marker for glioma stem cells (GSC)^53^. The Olig2 protein, which is mainly expressed in LGG, controls oligodendrocyte development in adult gliomas^54^. Ki-67 is a recognized predictive and prognostic indicator and is a widely used marker for assessing the mitotic index in tumors, including gliomas. Ki-67 expression in tumors is strongly associated with tumor cell proliferation and growth^55^. GFAP is expressed in mature astrocytes and certain astroglial cells of the central nervous system. In addition, neural stem cells also show strong GFAP expression. Because GFAP is highly expressed in mature astrocytes of healthy brain tissue, it is considered a marker of low-grade and highly differentiated tumors^56^. GFAP is usually present at higher concentrations in differentiated, slow-growing gliomas, while high GFAP expression can also be detected in malignancies^57^.

Synaptophysin, also known as p38 protein, is a major vesicular integral membrane protein specifically expressed in neuroendocrine tissues. The expression level of synptophysin in gliomas correlates with tumor grade and patient survival rate, the higher the tumor grade, the lower the expression level of synaptophysin decreases^58^. p53 is one of the key factors preventing neoplastic transformation. Physiologically, p53, encoded by the *TP53* gene, is a regulator of genes whose protein products are involved in processes such as cell cycle regulation, apoptosis and deoxyribonucleic acid (DNA) repair. Inhibition of p53 function, resulting from *TP53* gene mutations or epigenetic changes leading to its reduced expression, is associated with the progression of neoplastic disease, as well as resistance to radio- and chemotherapy^59,60^.

Other important factors involved in glioma pathogenesis are microRNAs (miRNAs). Among these, miR-155-5p has been reported to be overexpressed in various malignancies, including leukemia, esophageal cancer, prostate cancer, and gliomas^51,61^. Wu et al. demonstrated that the transfection of a miR-155 mimic in U87-MG glioma cells led to the downregulation of Caspase-3 and Caspase-9 mRNA levels, mediated by the activation of the anti-apoptotic Phosphoinositide 3-Kinase/Protein Kinase B (PI3K/AKT) signaling pathway, thereby promoting tumor cell proliferation^61^. Similarly, Guo et al. showed that increased miR-155-5p expression in U87 glioma cells reduced the levels of the pro-apoptotic protein BAX and simulateneously upregulated the anti-apoptotic protein B-cell lymphoma 2 (Bcl-2), whereas miR-155-5p silencing resulted in increased BAX and Caspase-3 expression^62^. Collectively, these findings indicate that miR-155-5p may negatively regulates apoptotic signaling in glioma cells, contributing to tumor progression and unfavorable clinical outcomes^51,61,62^. Importantly, miR-155-5p modulates PI3K/AKT signaling, which may additionally regulate the expression and activity of IAP family members^63^.

This study was conducted to evaluate the expression of IAP family genes in a homogeneous group of 26 patients diagnosed with pHGG. This is the first study aimed at evaluating the expression of individual *BIRC* family genes in correlation with p53, Olig2, Ki-67, and GFAP expression. Additionally, in the following study, we assessed the association of the expression of individual *BIRC* family genes with PD-1, mir-155-5p, survival rate and progression-free survival.

## Materials and methods

### Tumor sample collection and the approval of ethics committee

Tumor samples were collected from representative regions of the gliomas during neurosurgical resection in 26 pediatric patients hospitalized at the Children‘s Memorial Health Institute in Warsaw, Poland. The collected tissues were fixed in 4% phosphate-buffered formaldehyde. Secondary samples were embedded in paraffin, following standard procedure guidelines. All patients had a histopathological diagnosis performed by an experienced neuropathologist according to WHO 2016 classification (Grade IV glioblastoma). No Grade III anaplastic astrocytomas were included in the study cohort.

The age of patients (3–24 years, mean 11.9 ± 7.1) refers to their age at the moment of diagnosis; all of them were treated in a pediatric neuro-oncology unit. The characteristics of the studied patients depending on survival time and clinical parameters of the cancer are presented in Tables 2 and 3.

**Table 2:** Characteristics of the study group.

Parameter	N*	Mean	Median	Min	Max	SD	SE
Patient’s age	19	11.889	11.000	3.000	24.000	7.079	2.360
Survival rate	22	30.905	17.000	2.000	120.000	33.379	7.284
Overall survival	22	29.810	16.000	2.000	120.000	33.355	7.279
Progression free survival	22	27.429	14.000	2.000	120.000	34.080	7.437
mir 155-5p	26	27.929	2.189	0.325	118.056	40.827	8.007

*Not all parameters were available for the entire study group.

**Table 3:** Clinical parameters describing the study group.

Parameter	N*	%
PD-1		
0	6	23
1	2	8
2	3	12
3	2	8
Unknown	13	50
GFAP		
0	2	8
1	11	42
Unknown	13	50
Olig2		
0	10	38
1	3	12
Unknown	13	50
Ki67		
0	3	12
1	10	38
Unknown	13	50
p53		
0	8	31
1	5	19
Unknown	13	50
Synaptophysin		
0	12	46
1	1	4
Unknown	13	50

*Not all parameters were available for the entire study group.

n—number of cases; %—percentage distribution of the entire study cohort (*N* = 26).

PD-1 expression was evaluated semi-quantitatively as follows: 0—no staining; 1—weak staining (< 20% of positive cells); 2—moderate staining (20–40%); 3—strong staining (> 40%).

GFAP, Olig2, Ki-67, p53, and synaptophysin expression were evaluated dichotomously as follows: 0—no staining; 1—positive staining.

“Unknown” indicates cases for which immunohistochemical data were not available.

All patients included in the study received a diagnosis and treatment between 2013 and 2020. The study achieved full approval of the Medical University of Lublin Ethics Committee (KE-0254/330/2019). Due to the retrospective nature of the study and the use of archived, anonymized FFPE tissue samples, the requirement for informed consent was waived.

### Evaluation of immunohistochemistry findings

Immunohistochemical (IHC) staining for PD-1, GFAP, Olig2, Ki-67, p53, and synaptophysin was performed on formalin-fixed, paraffin-embedded (FFPE) pediatric high-grade glioma (pHGG) samples using the same protocol previously described by Litak et al.^51^.

Briefly, 4 μm sections were cut from FFPE tumor blocks using a microtome and mounted on Thermo Scientific™ SuperFrost™ Plus slides (Thermo Fisher Scientific, USA) to enhance cell adhesion.

Sections were deparaffinized and rehydrated through graded alcohols, followed by antigen retrieval in citrate buffer (pH 6.0) using the Dako Omnis IHC platform (Agilent, Santa Clara, CA, USA).

PD-1 expression was evaluated using the PD-L1 IHC 22C3 pharmDx kit (Agilent, Dako Omnis) according to the manufacturer’s instructions.

Other primary antibodies included anti-GFAP, anti-Olig2, anti-Ki-67, anti-p53, and anti-synaptophysin (all from Dako Omnis).

Signal visualization was performed with the EnVision™ Flex detection system (High pH; Agilent).

Tonsil tissue served as a positive control, and a Negative Control Reagent (Dako Omnis) was used for negative controls.

The stained slides were independently evaluated by two experienced pathologists.

Immunohistochemical staining for PD-1, GFAP, Olig2, Ki-67, p53, and synaptophysin was evaluated semi-quantitatively. PD-1 immunoreactivity was assessed using a four-tier scoring system as follows: 0—no detectable staining; 1—weak staining (< 20% of positive cells); 2—moderate staining (20–40%); and 3—strong staining (> 40%). For statistical analyses, PD-1 expression scores were grouped into low (scores 0–1) and high (scores 2–3) expression categories. GFAP, Olig2, Ki-67, p53, and synaptophysin expression were evaluated as binary variables (0—no staining; 1—positive staining).

Protein expression was classified as positive when ≥ 10% of tumor cells demonstrated specific nuclear or cytoplasmic staining.

### RNA isolation

Formalin-Fixed Paraffin-Embedded (FFPE) Tumors of pHGG were cut using a microtome into 10 μm slices. The Recover All Total Nucleic Acid Isolation Kit for FFPE Tissues (Fisher Scientific, Hampton, NH USA) was used for the total ribonucleic acid (RNA) enriched with a miRNA fraction isolation according to the manufacturer’s protocol. Isolated RNA was stored at − 80 °C until used.

The purity and concentration of RNA obtained from the FFPE was assessed using spectrophotometry (NanoDrop 2000c, ThermoFisher Scientific, Waltham, MA USA). Approximately 1 µg total RNA was used for complementary DNA (cDNA) synthesis. The reverse transcription process was performed according to the manufacturer’s protocol using the High-Capacity cDNA Reverse Transcription Kit with RNase Inhibitor (Applied Biosystem, Foster City, CA, USA) and the cDNA was stored at − 20 °C until used.

### Evaluation of the expression level of the studied genes

Quantitative real-time polymerase chain reaction (qPCR) was performed using the StepOnePlus™ Real-Time PCR System (Applied Biosystems, Waltham, MA, USA). The ΔΔCt method was used to determine the relative level of the expression of the examined gene^64^. Detailed procedures for RNA isolation and qPCR reactions are described in previously published papers^65–67^. The analysis of the obtained results was performed using Expression Suite Software 1.0.3 (Life Technologies).

GAPDH (Hs02786624_g1) was used as an endogenous control. GAPDH was selected as the reference gene based on its widespread use and reported stability in glioma and FFPE-derived tumor samples. In the analyzed cohort, no substantial variability in GAPDH Ct values was observed, supporting its suitability as an endogenous control.

The following TaqMan probes were used for the reaction: NAIP (Hs03037952_m1), BIRC2 (Hs00357350_m1), BIRC3 (Hs00154109_m1), XIAP (Hs00236913_m1), BIRC5 (Hs00153353_m1), BIRC6 (Hs00212288_m1), BIRC8 (Hs01057786), XAF1 (Hs01550138_m1), DIABLO (Hs00219876_m1), CASP3 (Hs00234387_m1) and CASP9 (Hs00962278_m1).

### Statistical analysis

Statistical analyses were performed using Statistica 13 software. Continuous variables were compared between groups using the Mann-Whitney U test, and correlations between them were assessed using the Spearman rank correlation coefficient. Overall survival (OS) and progression-free survival (PFS) were estimated using the Kaplan-Meier method, and differences between groups (divided into “high” and “low” expression relative to the median logRQ for *BIRC2* and *BIRC3*) were assessed using the log-rank test. Patients without a reported event were censored at the last available follow-up date. Due to the limited sample size and the exploratory nature of the study, multivariate regression models were not constructed. Univariate logistic regression models were used to determine the association between continuous expression levels (logRQ) of the *BIRC2/BIRC3* genes and dichotomous survival endpoints (OS and PFS > 12 and > 20 months). Results are presented as odds ratios (ORs) with 95% confidence intervals (95% CIs). To ensure consistent comparisons, regression analyses were restricted to the complete case set (patients with available data for both genes). Additional associations between markers and survival status and a sensitivity analysis for “early death” (OS < 9 months) were assessed using Fisher’s exact test; these results are provided in the supplementary materials. The threshold for statistical significance was *p* < 0.05.

Survival information was available for up to 22 patients; however, analyses combining survival endpoints with gene expression and/or immunohistochemical variables were restricted to the overlapping subset with complete biomarker data, therefore the effective N varied across survival analyses and is reported in the corresponding tables and figure legends.

## Results

Based on the conducted research, the presence of the transcript of the following genes: *BIRC2*, *BIRC3*, *BIRC5*, *BIRC6*, *BIRC7*, *CASP3*, *CASP9*, *DIABLO*, *NAIP*, *XAF1* and *XIAP* in the tumor tissue was demonstrated.

As part of the conducted research, the impact of clinical parameters (PD-1 expression, Olig2 expression, Ki67 expression, p53 expression in tumor cells and patient survival time) on the level of gene expression was assessed.

A number of positive moderate and strong correlations were demonstrated between the expression of genes examined in the tumor tissue. Moreover, it was observed that the gene expression: *BIRC2* (*r* = 0.59 *p* < 0.05), *BIRC3* (*r* = 0.536 *p* < 0.05), *CASP3* (*r* = 0.438 *p* < 0.05), *XAF1* (*r* = 0.443 *p* < 0.05) and *XIAP* (*r* = 0.561 *p* < 0.05) positively correlates with the level of mir155-5p in the tested material (Table 4).

**Table 4:** Correlations between the expression of tested genes and clinical parameters, * *p* < 0.05 spearman’s rank correlations.

Parameter	logRQ BIRC2	logRQ BIRC3	logRQ BIRC5	logRQ BIRC6	logRQ BIRC7	logRQ CASP3	logRQ CASP9	logRQ DIABLO	logRQ NAIP	logRQ XAF1	logRQ XIAP
*logRQ BIRC2*	1.000	0.584*	0.236	0.571*	0.191	0.485*	0.553*	0.623*	0.654*	0.677*	0.746*
*logRQ BIRC3*	0.584*	1.000	− 0.043	0.405	0.209	0.385	0.326	0.442*	0.291	0.525*	0.619*
*logRQ BIRC5*	0.236	− 0.043	1.000	0.431*	− 0.045	0.371	0.535*	0.539*	0.073	0.450*	0.377
*logRQ BIRC6*	0.571*	0.405	0.431*	1.000	0.673*	0.416*	0.768*	0.866*	0.634*	0.547*	0.766*
*logRQ BIRC7*	0.191	0.209	− 0.045	0.673*	1.000	− 0.036	0.555	0.600	0.136	0.555	0.491
*logRQ CASP3*	0.485*	0.385	0.371	0.416*	− 0.036	1.000	0.348	0.514*	0.158	0.533*	0.512*
*logRQ CASP9*	0.553*	0.326	0.535*	0.768*	0.555	0.348	1.000	0.793*	0.439*	0.582*	0.711*
*logRQ DIABLO*	0.623*	0.442*	0.539*	0.866*	0.600	0.514*	0.793*	1.000	0.498*	0.632*	0.803*
*logRQ NAIP*	0.654*	0.291	0.073	0.634*	0.136	0.158	0.439*	0.498*	1.000	0.352	0.537*
*logRQ XAF1*	0.677*	0.525*	0.450*	0.547*	0.555	0.533*	0.582*	0.632*	0.352	1.000	0.672*
*logRQ XIAP*	0.746*	0.619*	0.377	0.766*	0.491	0.512*	0.711*	0.803*	0.537*	0.672*	1.000
Age	− 0.464	0.071	− 0.036	− 0.357	− 0.400	0.086	− 0.286	− 0.179	0.071	− 0.143	− 0.321
PD− 1	0.245	0.345	0.085	− 0.123	− 0.202	0.657*	− 0.250	− 0.137	− 0.297	0.444	0.245
GFAP	− 0.100	− 0.300	− 0.300	− 0.500		0.058	− 0.500	− 0.500	− 0.500	− 0.200	− 0.400
Olig2	0.323	0.000	− 0.065	− 0.065	− 0.722	0.114	0.065	− 0.129	0.452	0.065	− 0.258
Ki67	− 0.373	− 0.596	0.149	− 0.224	0.000	0.174	− 0.224	− 0.149	− 0.671*	− 0.522	− 0.373
p53	0.231	− 0.115	− 0.115	− 0.346	− 0.474	0.174	0.000	− 0.173	0.289	0.346	− 0.462
Survival rate	− 0.478*	− 0.536*	0.202	0.108	0.262	− 0.177	0.184	0.014	− 0.224	− 0.272	− 0.127
Overall survival	− 0.458*	− 0.529*	0.191	0.124	0.381	− 0.182	0.189	0.018	− 0.227	− 0.250	− 0.116
Progression free survival	− 0.481*	− 0.540*	0.191	0.112	0.381	− 0.179	0.203	0.037	− 0.200	− 0.229	− 0.120
miR-155 5p	0.590*	0.536*	0.246	0.271	− 0.436	0.438*	0.310	0.315	0.331	0.443*	0.561*

### Dependence of the expression of the studied genes on the expression of PD-1 in tumor tissue

**Fig. 1:**
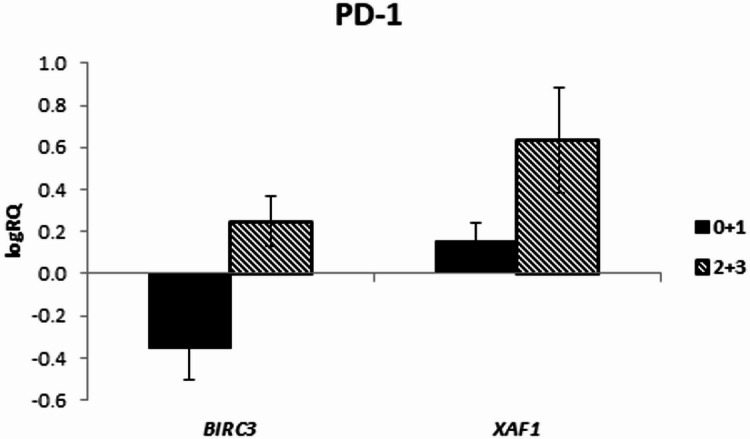
Mean (logRQ ± SE) expression of *BIRC3* and *XAF1* genes depending on PD-1 expression, The U Mann Whitney Test.

**Fig. 2:**
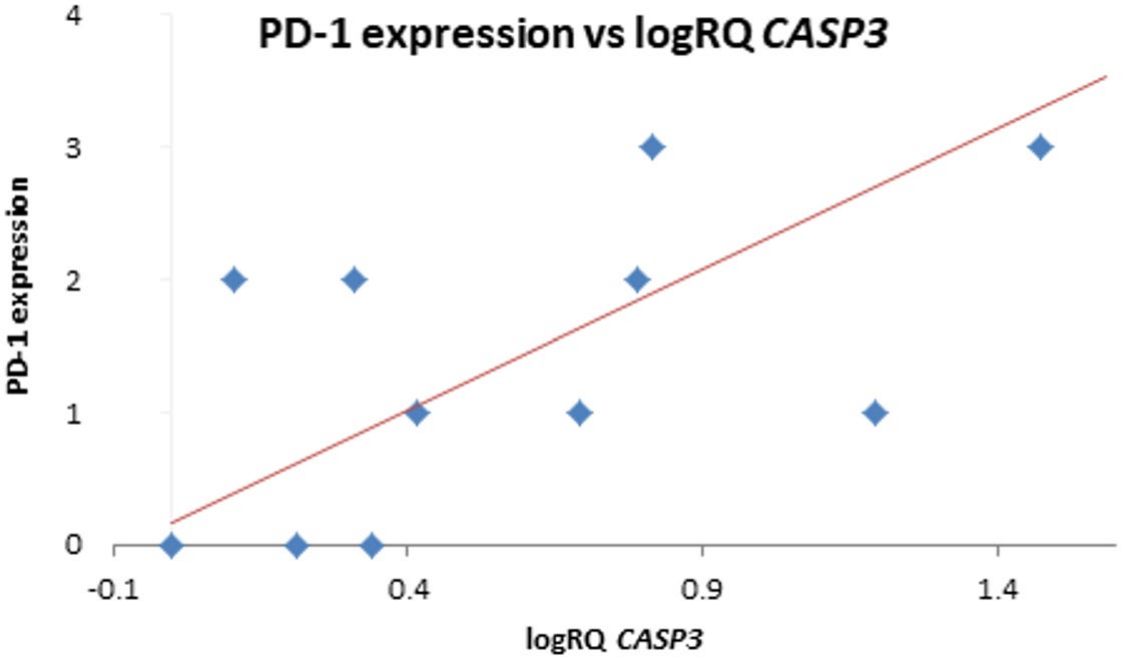
Scatterplot between *CASP3* gene expression (logRQ) and PD-1 expression (*r* = 0.657 *p* < 0.05 Spearman’s rank correlations).

The analysis of the relationship between the expression of the examined genes and the expression of PD-1 in the tumor tissue did not show any significant relationships; however, it was noted that in the case of the *BIRC3* and *XAF1* genes there is a tendency (*p* = 0.067 and *p* = 0.096 respectively) to higher levels of expression of these genes with higher PD-1 expression (Fig. 1; Table 5). In the correlation analysis, a strong positive relationship between *CASP3* gene expression and PD-1 expression was noted (*r* = 0.657 *p* < 0.05) (Table 4; Fig. 2).

**Table 5:** Mean expression of the examined genes depending on PD-1 expression, the U Mann Whitney Test.

Gene	PD-1 (0 + 1)			PD-1 (2 + 3)			*p*- value
	*N*	Mean	SD	*N*	Mean	SD	
PD-1							
*logRQ BIRC2*	8	− 0.142	0.401	5	− 0.005	0.228	0.516
*logRQ BIRC3*		− 0.355	0.494		0.248	0.456	**0.067**
*logRQ BIRC5*		0.472	0.340		0.374	0.466	0.696
*logRQ BIRC6*		− 0.159	0.417		− 0.274	0.296	0.619
*logRQ BIRC7*		− 0.089	0.848		− 0.314	0.284	0.634
*logRQ CASP3*		0.331	0.255		0.697	0.531	0.202
*logRQ CASP9*		0.078	0.379		− 0.239	0.235	0.140
*logRQ DIABLO*		− 0.317	0.470		− 0.385	0.332	0.790
*logRQ NAIP*		− 0.184	0.544		− 0.439	0.487	0.438
*logRQ XAF1*		0.150	0.399		0.635	0.717	**0.096**
*logRQ XIAP*		− 0.190	0.241		− 0.143	0.205	0.740

### Dependence of the expression of the studied genes on the expression of Olig2 in the tumor tissue

**Fig. 3:**
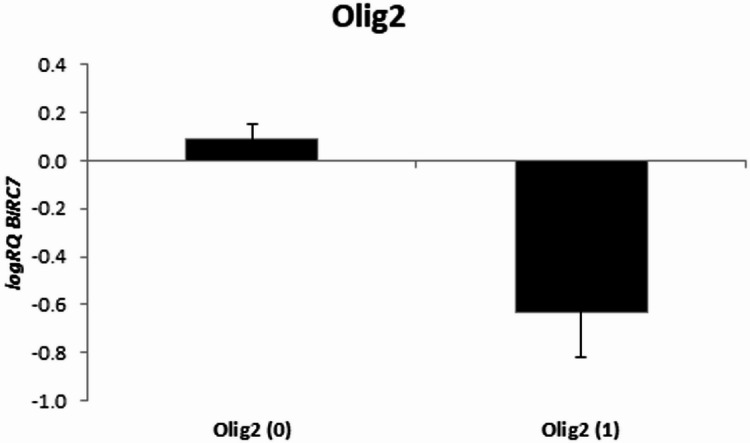
Mean (logRQ ± SE) expression of *BIRC7* gene depending on Olig2 expression, The U Mann Whitney Test.

The assessment of the relationship between Olig2 expression in tumor tissue and the expression of the examined genes did not show any significant relationships; however, a tendency (*p* = 0.072) toward lower *BIRC7* expression levels was noted in cases with positive Olig2 expression (Fig. 3; Table 6).

**Table 6:** Mean expression of the examined genes depending on Olig2 expression, (p-value, the U Mann Whitney Test)

Gene	Olig2 (0)			Olig2 (1)			*p*-value
	*N*	Mean	SD	*N*	Mean	SD	
Olig2							
*logRQ BIRC2*	10	− 0.139	0.362	3	0.079	0.144	0.351
*logRQ BIRC3*		− 0.098	0.494		− 0.037	0.816	0.881
*logRQ BIRC5*		0.459	0.318		0.342	0.608	0.677
*logRQ BIRC6*		− 0.182	0.418		− 0.287	0.061	0.686
*logRQ BIRC7*		0.093	0.465		− 0.631	0.333	**0.072**
*logRQ CASP3*		0.519	0.515		0.503	0.252	0.961
*logRQ CASP9*		− 0.069	0.394		− 0.059	0.252	0.970
*logRQ DIABLO*		− 0.322	0.447		− 0.417	0.265	0.744
*logRQ NAIP*		− 0.416	0.561		0.010	0.067	0.236
*logRQ XAF1*		0.337	0.561		0.460	0.792	0.777
*logRQ XIAP*		− 0.132	0.244		− 0.266	0.070	0.388

### Dependence of the expression of the studied genes on Ki67 expression in tumor tissue

**Fig. 4:**
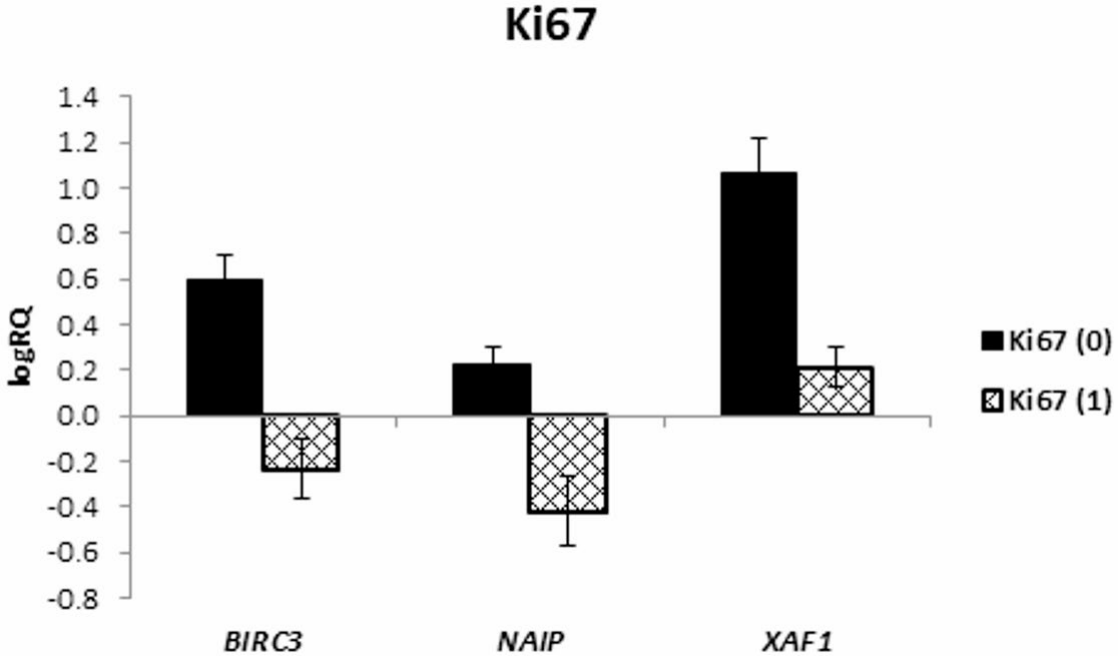
Mean (logRQ ± SE) expression of *BIRC3 (p = 0.049)*,* NAIP* (*p* = 0.008) and *XAF1* (*p* = 0.032) genes depending on Ki-67 expression, (The U Mann Whitney Test)

As a result of the analysis, the expression levels of *BIRC3* (*p* = 0.049), *NAIP* (*p* = 0.008), and *XAF1* (*p* = 0.032) were significantly higher in the tissues not expressing Ki-67 (Fig. 4), while the remaining genes did not show any significant correlations with Ki-67 expression (Table 7). The correlation analysis showed a negative relationship between *NAIP* gene expression and Ki-67 expression (*r* = − 0.671, *p* < 0.05) (Table 4). It should be noted, however, that the study group in which Ki-67 expression was not demonstrated included only 3 cases. Therefore, in order to confirm the obtained results, another analysis should be conducted on a larger group of patients.

**Table 7:** Mean expression of the examined genes depending on Ki-67 expression, (*p < 0.05 the U Mann Whitney Test)

Gene	Ki67 (1)			Ki67 (0)			*p*-value
	*N*	Mean	SD	*N*	Mean	SD	
Ki67							
*logRQ BIRC2*	10	− 0.127	0.343	3	0.133	0.101	0.334
*logRQ BIRC3*		− 0.231	0.485		0.592	0.243	0.049*
*logRQ BIRC5*		0.479	0.317		0.194	0.734	0.370
*logRQ BIRC6*		− 0.257	0.335		− 0.003	0.502	0.386
*logRQ BIRC7*		− 0.201	0.593		− 0.314	0.000	0.867
*logRQ CASP3*		0.562	0.482		0.323	0.023	0.523
*logRQ CASP9*		− 0.130	0.264		0.222	0.671	0.211
*logRQ DIABLO*		− 0.358	0.396		− 0.305	0.548	0.876
*logRQ NAIP*		− 0.418	0.484		0.231	0.203	0.008*
*logRQ XAF1*		0.215	0.514		1.071	0.424	0.032*
*logRQ XIAP*		− 0.194	0.222		− 0.054	0.196	0.436

### Dependence of the expression of the studied genes on the expression of p53 in tumor tissue

No significant correlations were found between the expression of the examined genes and p53 expression in tumor tissue (Table 8).

**Table 8:** Mean expression of the examined genes depending on p53 expression, (p-value, the U Mann Whitney Test)

Gene	p53 (0)			p53 (1)			*p*-value
	*N*	Mean	SD	*N*	Mean	SD	
p53							
*logRQ BIRC2*	8	-0.154	0.411	5	0.009	0.193	0.439
*logRQ BIRC3*		-0.018	0.494		-0.157	0.666	0.700
*logRQ BIRC5*		0.465	0.370		0.382	0.440	0.740
*logRQ BIRC6*		-0.114	0.465		-0.327	0.110	0.347
*logRQ BIRC7*		0.036	0.051		-0.319	0.629	0.485
*logRQ CASP3*		0.521	0.594		0.507	0.278	0.961
*logRQ CASP9*		-0.023	0.430		-0.119	0.257	0.672
*logRQ DIABLO*		-0.334	0.524		-0.365	0.215	0.903
*logRQ NAIP*		-0.456	0.638		-0.113	0.252	0.290
*logRQ XAF1*		0.209	0.556		0.565	0.633	0.346
*logRQ XIAP*		-0.083	0.263		-0.271	0.075	**0.099**

### Dependence of the expression of the studied genes on the survival rate

The expression of the examined genes was assessed depending on the survival rate in the groups below and above 20 months, as well as in the groups below and above 12 months.

**Fig. 5:**
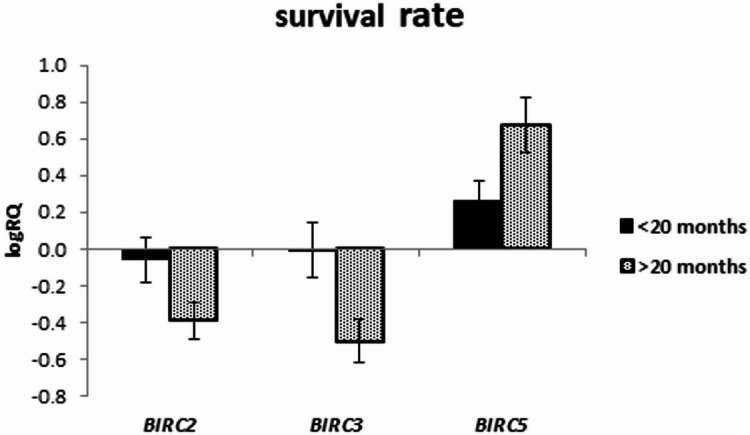
Mean (logRQ ± SE) expression of *BIRC2 (p = 0.025)*,* BIRC3* and *BIRC5* genes depending on survival rate (< 20 months and > 20 months), (The U Mann Whitney Test)

**Fig. 6:**
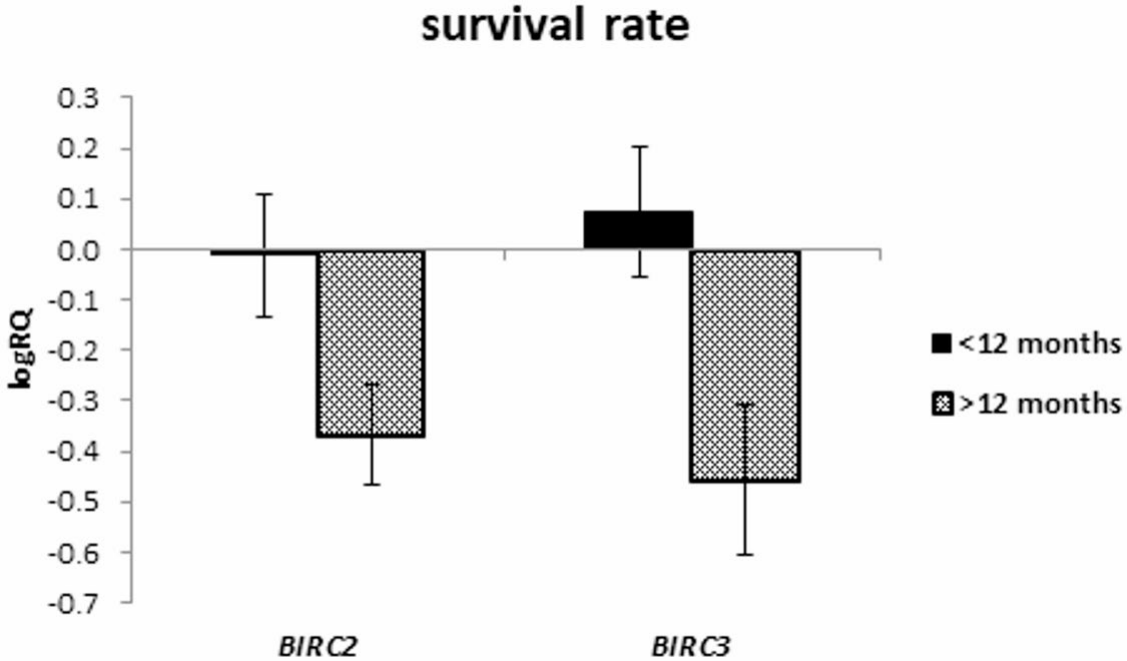
Mean (logRQ ± SE) expression of *BIRC2* and *BIRC3* genes depending on survival rate (< 12 months and > 12 months), (The U Mann Whitney Test)

**Fig. 7:**
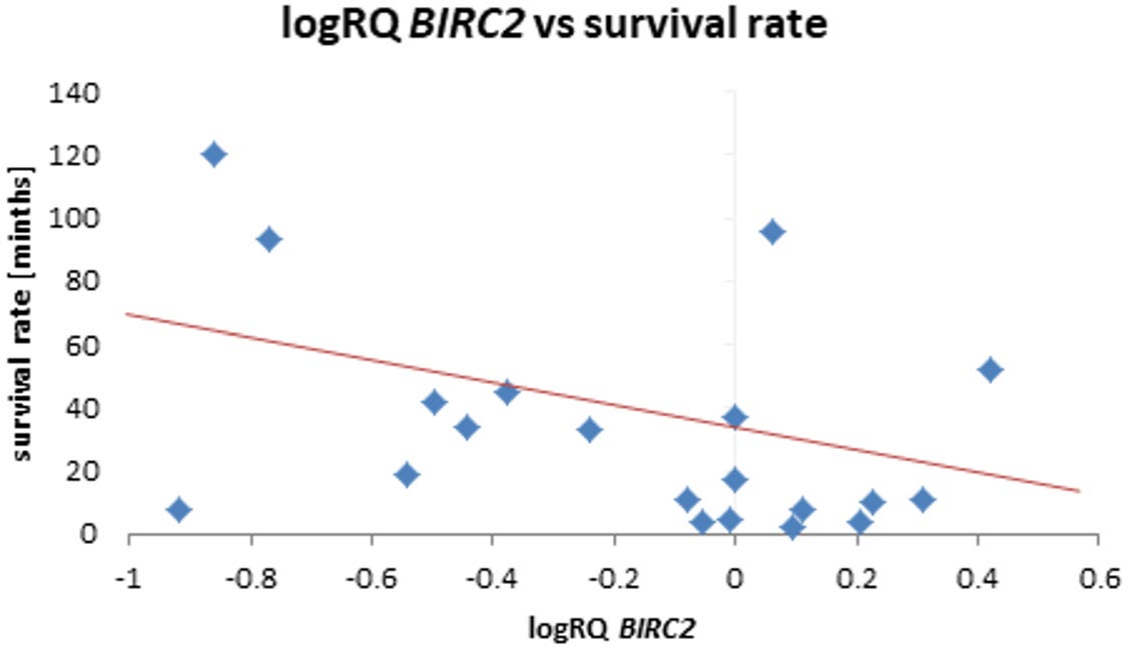
Scatterplot between *BIRC2* gene expression (logRQ) and survival rate (*r *= -0.478 *p* < 0.05 Spearman’s rank correlations).

**Fig. 8:**
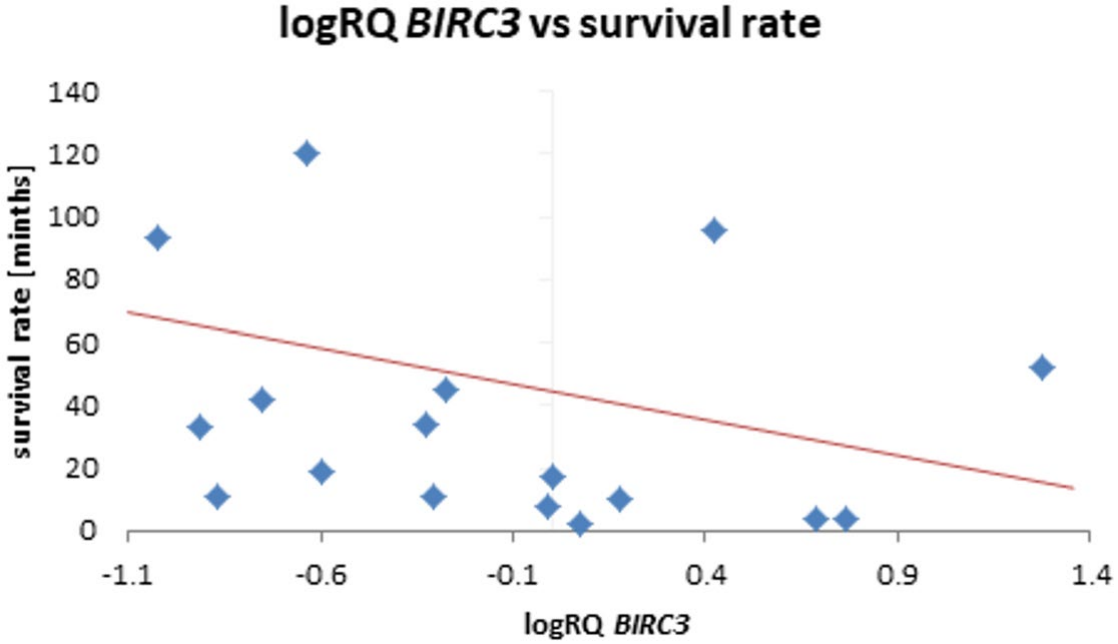
Scatterplot between *BIRC3* gene expression (logRQ) and survival rate (*r *= -0.536 *p* < 0.05 Spearman’s rank correlations).

The analysis of the relationship between the expression of the studied genes and the survival rate of 20 months showed a significantly higher expression of the *BIRC2* gene (*p* = 0.025) and a tendency (*p* = 0.080) to a higher expression value of the *BIRC3* gene in the group of patients whose survival time was shorter than 20 months (Fig. 5; Table 9). Similar results were obtained with a survival rate of 12 months, i.e. *BIRC2* gene expression was significantly higher (*p* = 0.037) in the tumor tissue of patients whose survival time was shorter than 12 months compared to those patients who lived longer. When assessing the *BIRC3* gene, a tendency (*p* = 0.057) to higher expression values with survival times shorter than 12 months was noted, close to statistical significance (Fig. 6; Table 10). The analysis of the correlation of survival time with the level of gene expression of the subjects showed significant negative correlations of *BIRC2* (*r*=-0.478 *p* < 0.05) and *BIRC3* (*r*=-0.536 *p* < 0.05) gene expression with the survival time index (Table 4; Figs. 7 and 8).

In turn, *BIRC5* gene expression showed the opposite tendency (*p* = 0.053), with higher values observed in patients whose survival time was longer than 20 months (Fig. 5; Table 9).

The remaining genes did not show significant relationships with the survival rate.

**Table 9:** Mean expression of the examined genes depending on survival rate (< 20 months and > 20 months), (*p < 0.05 the U Mann Whitney Test)

Gene	< 20 months		> 20 months		*p*-value
	Mean	SD	Mean	SD	
Survival rate					
*logRQ BIRC2*	− 0.060	0.362	− 0.391	0.329	0.025*
*logRQ BIRC3*	− 0.009	0.535	− 0.501	0.492	**0.080**
*logRQ BIRC5*	0.265	0.435	0.674	0.487	**0.053**
*logRQ BIRC6*	− 0.257	0.336	− 0.197	0.474	0.748
*logRQ BIRC7*	− 0.461	0.362	− 0.455	0.669	0.987
*logRQ CASP3*	0.437	0.283	0.377	0.587	0.774
*logRQ CASP9*	− 0.229	0.337	− 0.107	0.404	0.485
*logRQ DIABLO*	− 0.379	0.416	− 0.425	0.466	0.825
*logRQ NAIP*	− 0.365	0.491	− 0.540	0.673	0.519
*logRQ XAF1*	0.214	0.678	0.148	0.697	0.838
*logRQ XIAP*	− 0.219	0.274	− 0.283	0.403	0.682

**Table 10:** Mean expression of the examined genes depending on survival rate (< 12 months and > 12 months), (*p < 0.05 the U Mann Whitney Test)

Gene	> 12 months		< 12 months		*p*-value
	Mean	SD	Mean	SD	
Survival rate					
*logRQ BIRC2*	− 0.367	0.321	− 0.013	0.364	0.037*
*logRQ BIRC3*	− 0.456	0.461	0.074	0.562	**0.057**
*logRQ BIRC5*	0.486	0.598	0.383	0.364	0.662
*logRQ BIRC6*	− 0.248	0.482	− 0.213	0.278	0.850
*logRQ BIRC7*	− 0.342	0.592	− 0.577	0.293	0.503
*logRQ CASP3*	0.345	0.525	0.483	0.271	0.494
*logRQ CASP9*	− 0.163	0.417	− 0.194	0.311	0.860
*logRQ DIABLO*	− 0.435	0.469	− 0.358	0.395	0.702
*logRQ NAIP*	− 0.543	0.649	− 0.323	0.463	0.411
*logRQ XAF1*	0.046	0.673	0.342	0.664	0.350
*logRQ XIAP*	− 0.300	0.396	− 0.186	0.232	0.463

### Dependence of the expression of the studied genes on progression-free survival

**Fig. 9:**
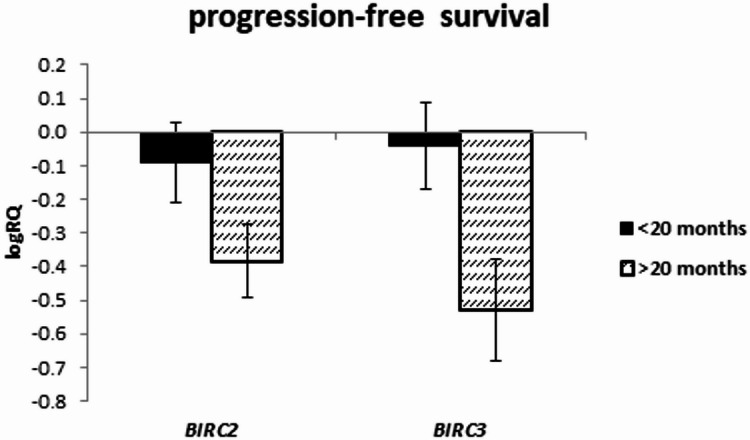
Mean (logRQ ± SE) expression of *BIRC2* and *BIRC3* genes depending on progression free survival (< 20 months and > 20 months), (The U Mann Whitney Test)

**Fig. 10:**
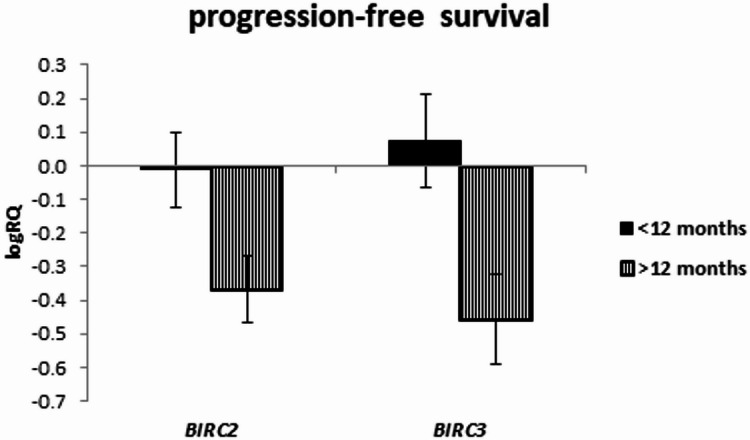
Mean (logRQ ± SE) expression of *BIRC2* and *BIRC3* genes depending on progression-free survival (< 12 months and > 12 months), (The U Mann Whitney Test)

**Fig. 11:**
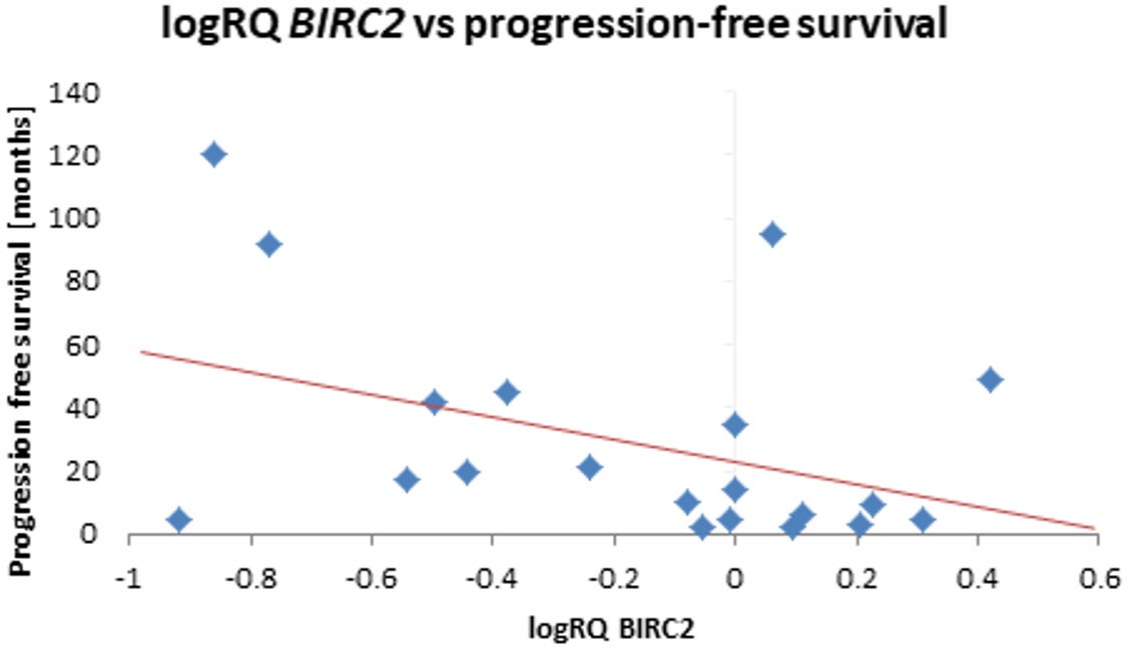
Scatterplot between *BIRC2* gene expression (logRQ) and progression-free survival (*r*=-0.481 *p* < 0.05 Spearman’s rank correlations).

**Fig. 12:**
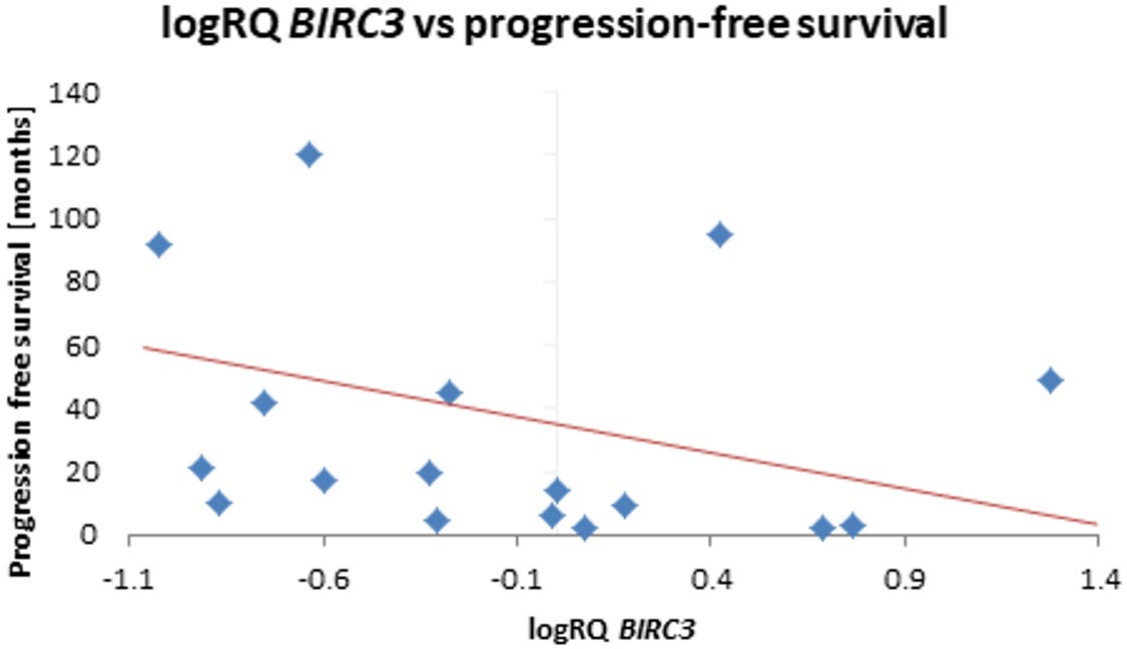
Scatterplot between *BIRC3* gene expression (logRQ) and progression-free survival (*r*=-0.536 *p* < 0.05 Spearman’s rank correlations).

An analysis of gene expression was performed depending on progression-free survival, with the cut-off being 12 months and 20 months. It has been noted that the expression of *BIRC2* and *BIRC3* genes tends to be higher with progression-free survival shorter than 20 months (Fig. 9; Table 11). If the 12-month limit was used, it was observed that *BIRC2* expression in the tumor tissue of patients with progression-free survival shorter than 12 months is significantly higher. In the case of *BIRC3* gene, there is a tendency (*p* = 0.057) to higher expression values with progression-free survival shorter than 12 months (Fig. 10; Table 12). A correlation analysis of the examined gene expression and progression-free survival showed a significant negative relationship with *BIRC2* gene expression (*r*=-0.481 *p* < 0.05) and *BIRC3* gene expression (*r*=-0.540 *p* < 0.05) in the tumor tissue (Table 4; Figs. 11 and 12).

The remaining genes did not show significant relationships with progression-free survival.

**Table 11:** Mean expression of the examined genes depending on progression-free survival (< 20 months and > 20 months), (*p-value, the U Mann Whitney Test)

Gene	< 20 months		> 20 months		*p*-value
	Mean	SD	Mean	SD	
Progression-free survival (PFS)					
*logRQ BIRC2*	− 0.092	0.363	− 0.384	0.354	**0.068**
*logRQ BIRC3*	− 0.040	0.515	− 0.530	0.533	**0.090**
*logRQ BIRC5*	0.338	0.485	0.607	0.486	0.259
*logRQ BIRC6*	− 0.266	0.322	− 0.173	0.507	0.631
*logRQ BIRC7*	− 0.586	0.445	− 0.079	0.215	0.186
*logRQ CASP3*	0.436	0.270	0.370	0.643	0.761
*logRQ CASP9*	− 0.238	0.323	− 0.074	0.424	0.354
*logRQ DIABLO*	− 0.372	0.397	− 0.445	0.500	0.730
*logRQ NAIP*	− 0.378	0.471	− 0.542	0.727	0.557
*logRQ XAF1*	0.191	0.651	0.179	0.747	0.972
*logRQ XIAP*	− 0.229	0.263	− 0.275	0.434	0.774

**Table 12:** Mean expression of the examined genes depending on progression-free survival (< 12 months and > 12months), (*p < 0.05, the U Mann Whitney Test)

Gene	> 12 months		< 12 months		*p*-value
	Mean	SD	Mean	SD	
Progression-free survival					
*logRQ BIRC2*	− 0.367	0.321	− 0.013	0.364	0.037*
*logRQ BIRC3*	− 0.456	0.461	0.074	0.562	**0.057**
*logRQ BIRC5*	0.486	0.598	0.383	0.364	0.662
*logRQ BIRC6*	− 0.248	0.482	− 0.213	0.278	0.850
*logRQ BIRC7*	− 0.342	0.592	− 0.577	0.293	0.503
*logRQ CASP3*	0.345	0.525	0.483	0.271	0.494
*logRQ CASP9*	− 0.163	0.417	− 0.194	0.311	0.860
*logRQ DIABLO*	− 0.435	0.469	− 0.358	0.395	0.702
*logRQ NAIP*	− 0.543	0.649	− 0.323	0.463	0.411
*logRQ XAF1*	0.046	0.673	0.342	0.664	0.350
*logRQ XIAP*	− 0.300	0.396	− 0.186	0.232	0.463

### Logistic regression analysis for survival and disease progression

**Fig. 13:**
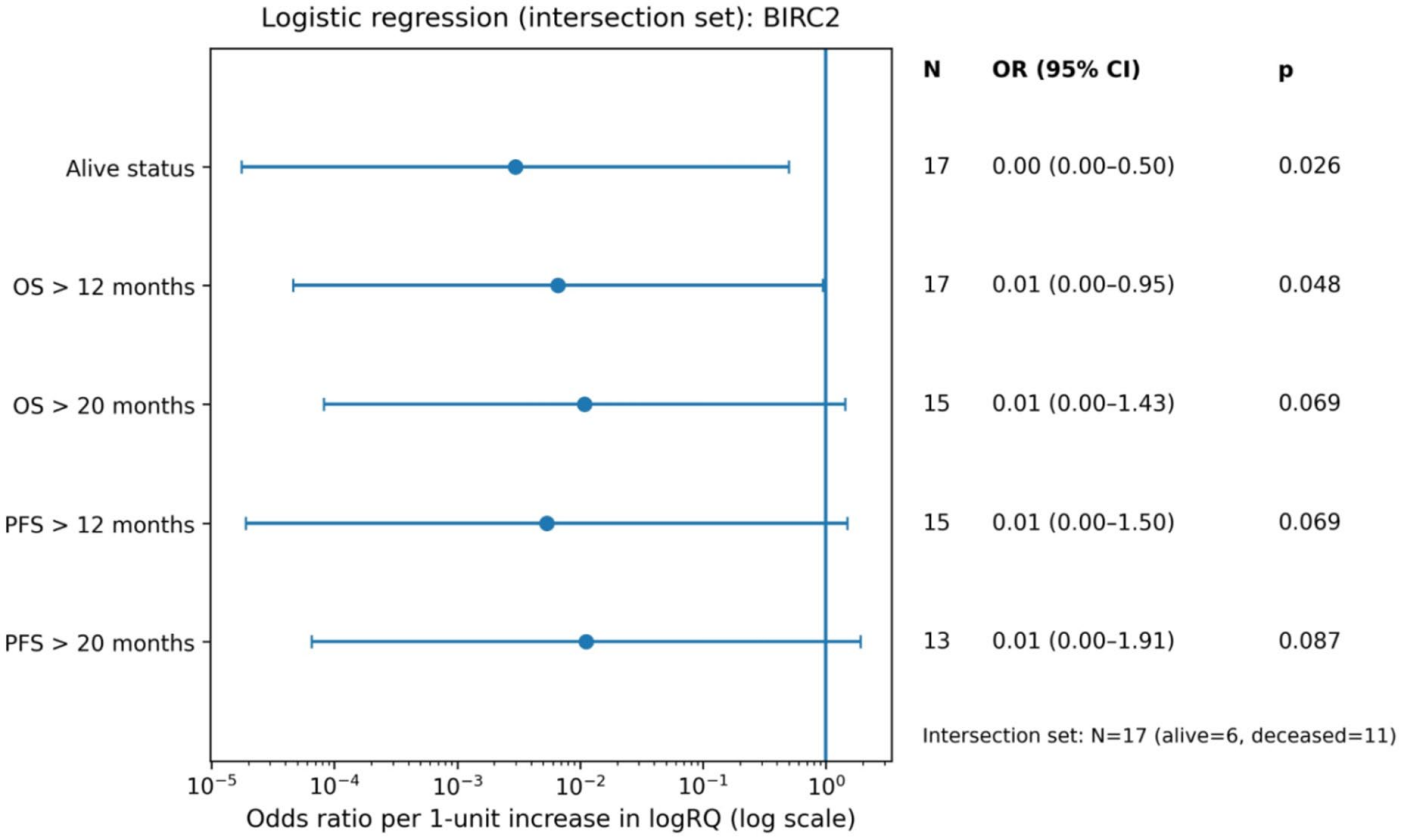
Forest plot of odds ratios (OR) from univariate logistic regression for dichotomized survival endpoints (OS/PFS) in the intersection set, using *BIRC2* expression (logRQ) as predictor.

**Fig. 14:**
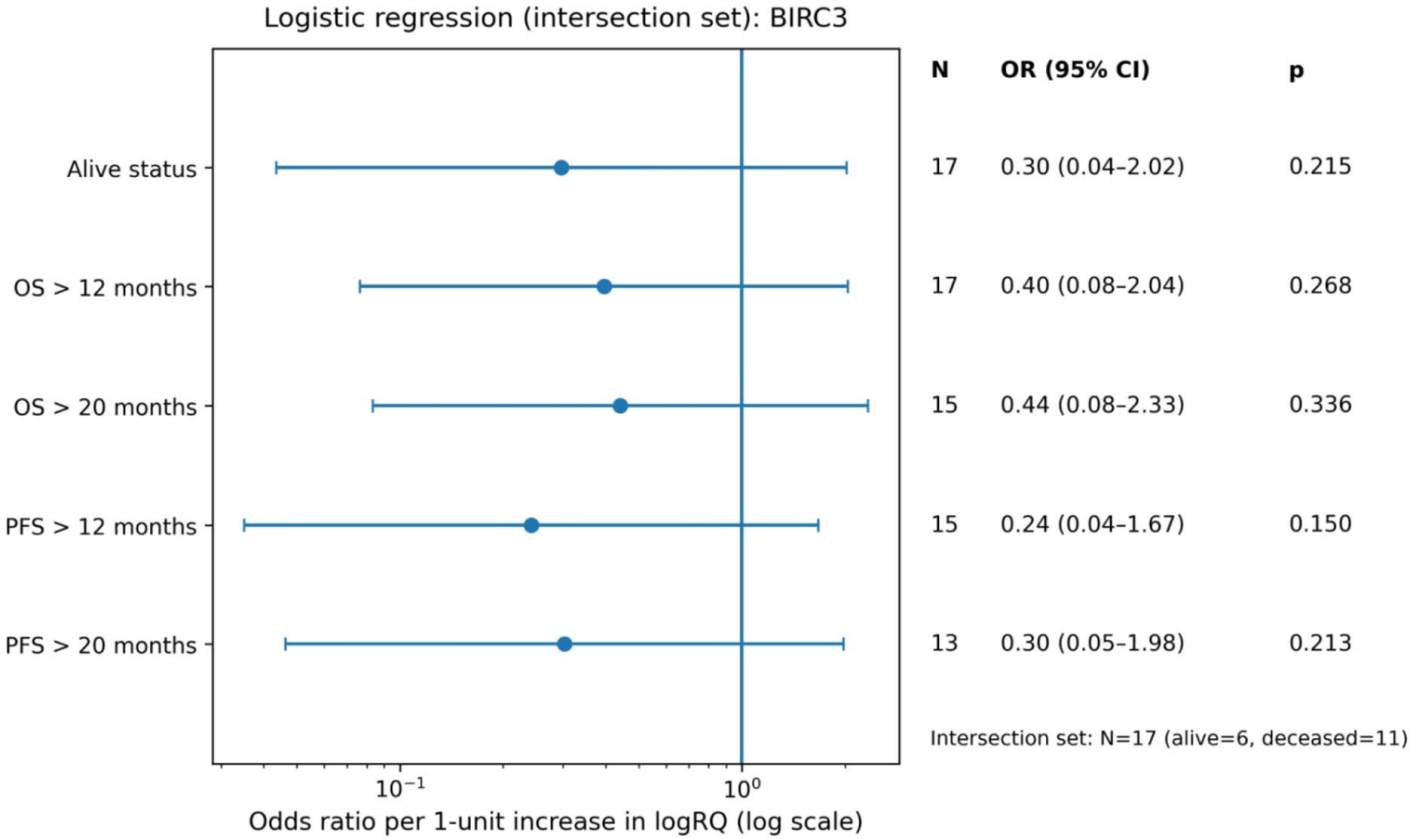
Forest plot of odds ratios (OR) from univariate logistic regression for dichotomized survival endpoints (OS/PFS) in the intersection set, using *BIRC3* expression (logRQ) as predictor.

Univariate logistic regression models assessed the effect of continuous *BIRC2* and *BIRC3* expression levels (logRQ) on the probability of achieving long-term survival endpoints (Supplementary). The analysis showed that *BIRC2* expression level was a statistically significant predictor of survival beyond 12 months (OR = 0.01; 95% CI: 0.00–0.95; *p* = 0.048). This indicates that increased *BIRC2* expression was associated with significantly lower odds of achieving this survival threshold. Similar trends were observed for the remaining endpoints for *BIRC2*, where the results were close to statistical significance: OS > 20 months (*p* = 0.069), PFS > 12 months (*p* = 0.069), and PFS > 20 months (*p* = 0.087), with consistently low odds ratios (OR = 0.01) (Fig. 13). For *BIRC3*, although the OR values also suggested a negative trend (OR range 0.24–0.44), none of the analyzed associations reached statistical significance (*p* > 0.05) (Fig. 14).

**Fig. 15:**
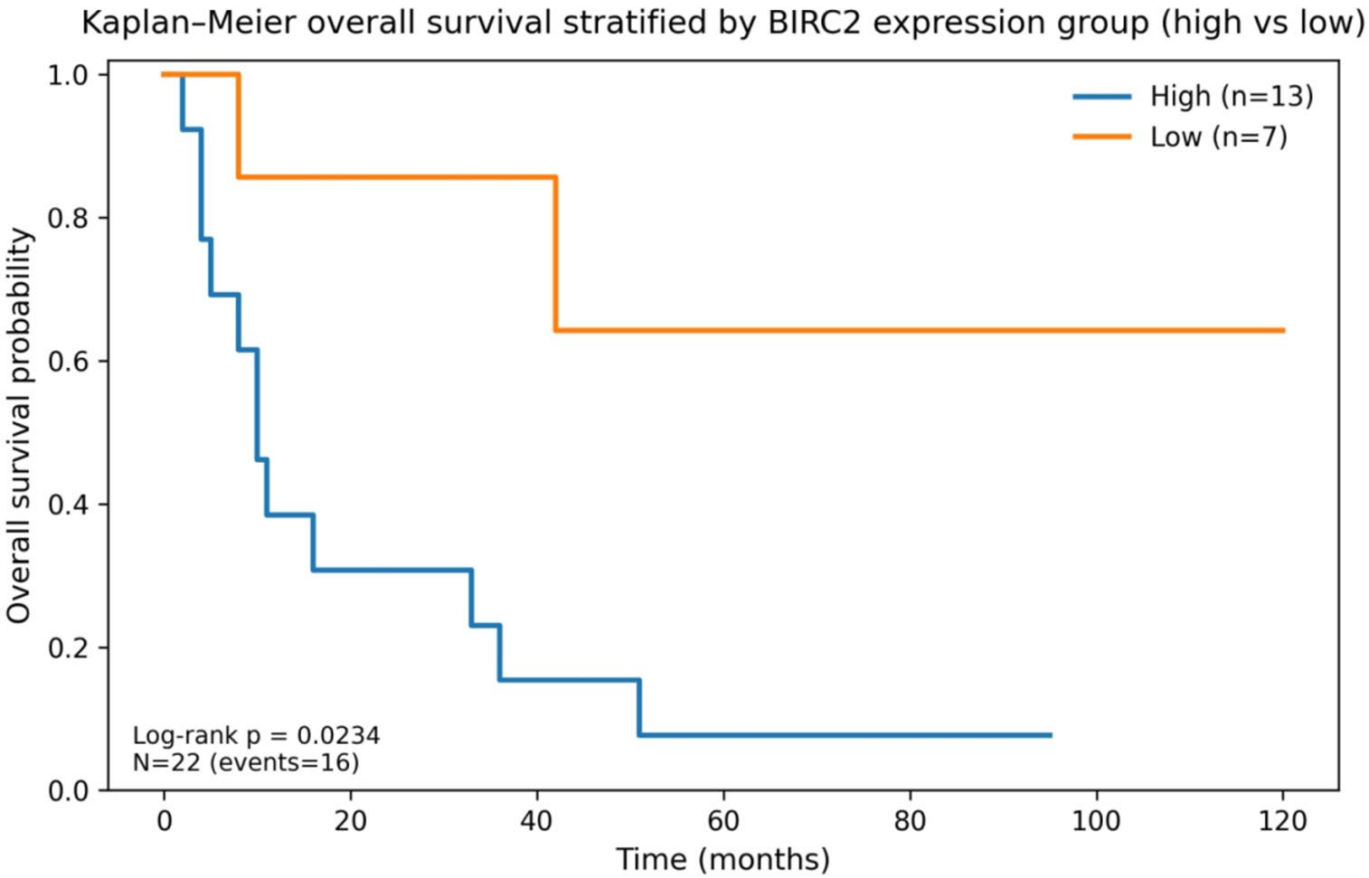
Kaplan–Meier overall survival stratified by *BIRC2* expression group (high vs. low). Log-rank *p* = 0.023* *BIRC2* group (high = logRQ*BIRC2*>-0.077794 (median) /low = logRQ*BIRC2*<=-0.077794).

**Fig. 16:**
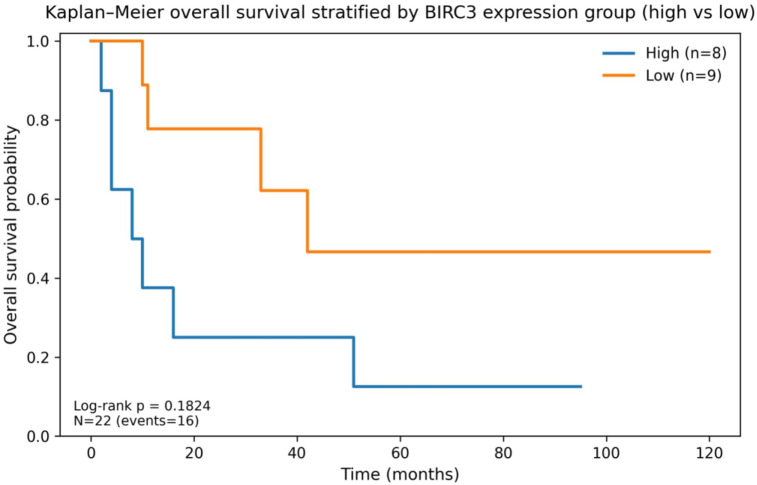
Kaplan–Meier overall survival stratified by *BIRC3* expression group (high vs. low). Log-rank *p* = 0.1824. *BIRC3* group (high = logRQ*BIRC3*>-0.316210 (median) /low = logRQ*BIRC3*<=-0.316210).

Kaplan–Meier analyses showed a significant difference in OS between *BIRC2* high vs. low groups (log-rank *p* = 0.023) (Fig. 15) and no significant difference in OS between *BIRC3* high vs. low groups (log-rank *p* = 0.182) (Fig. 16). PD-1 high expression (score 2–3) was associated with shorter OS compared with PD-1 low (score 0–1) with borderline significance (log-rank *p* = 0.082). In an exploratory early-death analysis (OS < 9 months), PD-1 high was enriched among early deaths (Fisher’s exact *p* = 0.007) (Supplementary).

Despite the lack of statistical significance in some of the survival analyses, they did reveal some molecular trends. Due to the pilot nature of these observations and the limited sample size, it was decided to present them in the supplementary materials to ensure full data transparency.

### Dependence of the expression of the studied genes on gender or age of the patients

No significant correlations between the expression of the studied genes in the tumor tissue and the gender or age of the patients were observed.

## Discussion

In our study, we analyzed the expression of IAP family genes and their regulators in 26 pediatric patients diagnosed with pHGG and evaluated the relationship between the available clinical data and the expression levels of the studied genes. To the best of our knowledge, this is the first study to assess the relationship between the expression of genes of all IAP family and their regulators in such a homogeneous group of patients diagnosed with pHGG. Previous studies have mainly focused on adult HGG, and their results were often extrapolated to pHGG^68–71^. Although several referenced studies concern adult high-grade gliomas and other adult tumors, pediatric high-grade gliomas represent a biologically distinct entity with unique molecular and clinical characteristics. Therefore, findings from adult tumors were used primarily to provide contextual background and mechanistic hypotheses, rather than as direct evidence for pediatric disease. Interpretation of the present results was based primarily on data derived from the pediatric cohort analyzed in this study.

It should be emphasized that all associations identified in this study are correlative in nature. Given the limited sample size, causal relationships cannot be inferred.

Our research demonstrated the presence of mRNA transcripts of *BIRC2*,* BIRC3*,* BIRC5*,* BIRC6*,* BIRC7*,* CASP3*,* DIABLO*,* NAIP*,* XAF1* and *XIAP* genes in pHGG cells. This is consistent with previous studies on gliomas^72–76^. The expression of IAPs has been demonstrated in many other types of cancertheir increased expression being usually associated with an unfavorable course of the disease^15,17–23,77^.

The expression of *BIRC2* and *BIRC3* has also been shown to be associated with the phenomenon of adaptive resistance to immunotherapy, limiting the efficacy of PD-1 blockade. Sehgal et al. identified a small subpopulation of immunotherapy-persistent tumor cells that were resistant to CD8^+^-mediated killing. This population was susceptible to tumor necrosis factor-α-induced cytotoxicity, and the silencing of *BIRC2* and *BIRC3* expression combined with the use of PD-1 therapy enhanced the destruction of cancer cells^78^. In our study, we showed that there is a negative correlation between PD-1 expression in tumor tissue samples and survival time, which may be linked to a relationship between PD-1 and tumor aggressiveness. At the same time, we observed that in the case of high PD-1 expression in tumor tissue, there is a tendency (*p* = 0.067) toward higher levels of *BIRC3* gene expression, which is associated with unfavorable prognosis.

In a study by Biswas et al., cIAP2-/- mice showed increased severity of experimental autoimmune encephalomyelitis, greater CD4^+^ T cell infiltration, higher expression of pro-inflammatory cytokines/chemokines and enhanced demyelination. The authors suggest that cIAP2 is required to limit disease severity and associated neuroinflammation^79^. In relation to our results, it can be assumed that increased expression of *BIRC3* in cells with high PD-1 expression enhances the phenomenon of tumor evasion from immune detection and destruction by reducing the inflammation in the glioma microenvironment.

Moreover, our analysis of the correlation between mir-155-5p concentration and the studied genes showed positive correlations with the expression level of *BIRC2*, *BIRC3*, *CASP3*, *XAF1* and *XIAP*. This may be related to the regulation of the PI3K/AKT signalling pathway by miR-155-5p^61^. The activation of the PI3K/AKT pathway, via NF-κB, may increase the transcription of anti-apoptotic genes, such as *BIRC2*, *BIRC3*, and *XIAP*^63^. Also, in our previous work, we showed significant correlations of mir-155-5p with PD-1, suggesting that miR-155-5p may not only modulate apoptosis in glioma cells but also contribute to modulating the immunosuppressive tumor microenvironment^51^.

As mentioned earlier, while assessing the expression level of *BIRC2* and *BIRC3* genes in relation to survival time from diagnosis, we noted. The higher expression of the *BIRC2* gene and a tendency for the higher expression of the *BIRC3* gene in the group of patients whose survival time was shorter than 20 months. We obtained similar results using the survival rate of 12 months. In this case, we noted the significantly higher expression of the *BIRC2* gene and a tendency (*p* = 0.057) close to statistical significance for the higher expression values of the *BIRC3* gene.

In the case of the analysis of *BIRC2* and *BIRC3* gene expression depending on progression-free survival, we observed a tendency (*p* = 0.080) for higher expression values of both genes with progression-free survival shorter than 20 months. Similar results were obtained when the threshold of progression-free survival was set at 12 months. In this case, it was observed that *BIRC2* expression in the tumor tissue is significantly higher in these patients, while in the case of *BIRC3* gene expression we observed a tendency for higher expression values in patients with progression-free survival shorter than 12 months.

In this single-center pediatric HGG cohort, applying predefined logRQ cut-offs for dichotomization indicated that high *BIRC2* expression was associated with shorter overall survival in Kaplan–Meier analysis. In contrast, *BIRC3* did not show a significant OS difference between expression groups. We performed also exploratory univariate logistic regression using continuous logRQ values for dichotomized OS/PFS endpoints. The analysis showed that *BIRC2 *expression level was a significant predictor of survival beyond 12 months.

Given the limited sample size and missing expression data for a subset of cases, these findings should be interpreted as hypothesis-generating and require external validation.

The results we obtained are consistent with the data published by Gressot et al. The authors in an examination of the cancer genome atlas expression data, reported that - among all described IAPs - only *BIRC3* both correlated with shorter survival in both LGG and GB and exhibited increased expression in GB relative to LGGs. They showed that, out of eight known IAPs, *BIRC3* has a unique role in facilitating glioma progression from low- to high-grade^74^. Wu et al. showed that high *BIRC3* expression can significantly promote tumor initiation and progression, and furthermore, *BIRC3* depletion can increase survival by inhibiting tumor initiation and growth^80^. Additionally, their analysis indicates that *BIRC3* is an independent stem biomarker in patient-derived GSCs. Data presented by Wu et al. indicate that *BIRC3* can significantly enhance the ability of the tumor to form neurospheres in both human or murine GBM and patient-derived GSC cell lines^80^. Wang et al. showed that patients with downregulated expressions of *BIRC3* demonstrated a significantly enhanced overall survival compared to patients with higher *BIRC3* expression^81^. Furthermore, using patient tissue samples, they showed that *BIRC3* expression increases with the recurrence of the disease, and additionally they demonstrated an increase in *BIRC3* expression in response to irradiation and temozolomide treatment. Finally, they showed evidence that the upregulation of *BIRC3* causes apoptosis evasion and therapeutic resistance in GBM^81^.

### Limitations of the study

It should be emphasized, however, that our study has several limitations. Firstly, we examined a relatively small group of patients. Although the cohort was homogeneous, the limited sample size impacts the statistical power of the study. Another limitation is the limited availability of pediatric-specific reference studies, which necessitated cautious comparison with data derived from adult tumors. Due to the limited sample size, regression analyses were not performed, as they would lack sufficient statistical power and could lead to overinterpretation of the results. Secondly, we did not obtain clinical data on the expression of PD1, GFAP, Olig2, Ki67, p53 and synaptophysin for all patients. Therefore, analyses including these parameters were performed on smaller subsets, which may have introduced bias and limited the robustness of the statistical associations. Consequently, these analyses should be repeated in a larger cohort. Information on survival time was available for 22 of 26 patients, and significant results were obtained for the group of 22 patients with pHGG. Additionally, we examined the expression of genes from the IAP family and their regulators at the mRNA level, whereas protein-level analyses were not performed. This limits the ability to determine whether the observed changes in mRNA expression translate into functional differences at the protein level. Therefore, the observed associations should not be interpreted as evidence of causal relationships. A matched non-neoplastic brain tissue was unavailable for ethical reasons; therefore, expression levels were analyzed in relation to the cohort median. Results were normalized to GAPDH, the values reflecting intra-cohort differences rather than direct fold-changes versus the healthy tissue. As only mRNA levels were assessed, future studies should include larger cohorts and protein-level analyses to validate these findings.

## Conclusions

To summarize, our study may reflect that the expression of IAP family genes and their regulators - *NAIP*, *BIRC2*, *BIRC3*, *XIAP*, *BIRC5*, *BIRC6*, *BIRC7*, *CASP3*, *CASP9*, *DIABLO* and *XAF1* may be associated with selected clinical parameters, including PD-1 expression, Olig2 expression, Ki67 expression, p53 expression in tumor cells, overall survival and progression-free survival.

We found that the expression of *BIRC3*, *NAIP* and *XAF1* was significantly higher in tumor tissues not expressing Ki-67, while the remaining genes did not show any significant correlations with Ki-67 expression. In addition, *BIRC2* and *BIRC3* expression levels showed significant negative correlations with overall survival and progression-free survival time. Overall, our results suggest that higher *BIRC2* expression may have prognostic relevance in pediatric high-grade glioma, whereas evidence for *BIRC3* was not conclusive in the present cohort. These observations warrant confirmation in larger, independent datasets with harmonized molecular and clinical annotation.

Moreover, we noticed that higher expression levels of *BIRC3* and *XAF1* in tumor tissue were accompanied by higher PD-1 expression. A strong positive correlation between *CASP3* expression and PD-1 expression was also noted. Additionally, a tendency (*p* = 0.072) toward lower *BIRC7* expression levels was observed in Olig2-positive tumors. The assessment of the relationship between p53 expression in tumor tissue and the expression of the examined genes did not show any significant relationships.

To the best of our knowledge, this is the first study to assess the relationship between expression of the entire IAP family and their regulators in a homogeneous group of patients diagnosed with pHGG. Our findings may contribute to a better understanding of molecular mechanisms involved in the pathogenesis of pHGGs, but they should be interpreted with caution and confirmed in larger, more detailed studies. These findings should be considered exploratory and hypothesis-generating rather than confirmatory.

Taken together, our study observed in association with that *BIRC2* and *BIRC3* may play a key role in apoptosis resistance mechanisms and immunomodulatory networks in pHGG, potentially contributing to aggressive disease progression and poor treatment outcomes. Their association with poor survival suggests that IAP family members may be prognostic biomarkers reflecting tumor aggressiveness. Furthermore, the observed associations between IAP expression, checkpoint activation, and clinical outcomes suggest that tagging apoptosis resistance mechanisms could enhance the efficacy of current treatment strategies. Despite the aforementioned limitations, these results warrant further investigation of IAP family members and miR-155-5p as potential prognostic biomarkers and therapeutic targets in pHGG.

## Supplementary Information

Below is the link to the electronic supplementary material.

Supplementary Material 1

## Data Availability

The data used to support the findings of this study are included in the article.
