# Supplementary material for: Reduced expression of BIRC2 and BIRC3 associated with longer survival in pediatric high-grade gliomas
Source: Sci Rep. 2026 Jan 30;16:6665. doi: 10.1038/s41598-026-35887-7 (PMC12913816; doi:10.1038/s41598-026-35887-7)
Supplement: Supplementary file 1 — Supplementary Material 1 [file 41598_2026_35887_MOESM1_ESM.docx]

**Methods**

Overall survival (OS) and progression-free survival (PFS) were summarized using the Kaplan–Meier method. Patients without an observed event were censored at the last available follow-up time recorded in the dataset. Group comparisons (high vs low) were assessed using the log-rank test. Given the limited sample size and the exploratory nature of this study, multivariable regression models were not performed. Additionally, exploratory univariate logistic regression models were fitted to quantify associations between BIRC2/BIRC3 expression (logRQ, continuous) and dichotomized survival endpoints (OS >12 and >20 months; PFS >12 and >20 months), reporting odds ratios (OR) with 95% confidence intervals (95% CI). To ensure comparable sample sizes between BIRC2 and BIRC3, these regression analyses were restricted to the complete-case intersection set (patients with non-missing BIRC2 and BIRC3). Univariate associations between marker groups and survival status were evaluated using Fisher’s exact test and are provided as supplementary results. A sensitivity analysis based on an ‘early death’ definition (OS < 9 months) was additionally performed using Fisher’s exact test.

* Because biomarker expression and immunohistochemical data were missing for some patients, survival analyses were performed on complete cases for the variables included; therefore, N differs between analyses and is indicated in each table/figure

**Result**

**Supplementary Table 1. Baseline characteristics**

| **Characteristic** | **N (22)** |
| --- | --- |
| Deaths, n (%) | 16 (72.7%) |
| Censored/alive at last follow-up, n (%) | 6 (27.3%) |
| BIRC2 group (high/low) | 11/11 |
| BIRC3 group (high/low) [non-missing] | 8/9 (missing: 5) |
| PD-1 group (high/low) [non-missing] | 5/8 (missing: 9) |
| Overall survival (months), median (range) | 16.5 (2.0–120.0) |
| Progression-free survival (months), median (range) | 15.5 (2.0–120.0) |

BIRC2 group (high= logRQBIRC2>-0.077794 (median) /low= logRQBIRC2<=-0.077794),
BIRC3 group (high= logRQBIRC3>-0.316210 (median) /low= logRQBIRC3<=-0.316210),

PD-1 group (low=0–1, high=2–3)

**Supplementary Table 2. Kaplan–Meier survival comparisons (OS)**

| **Marker** | **Group** | **N** | **Events** | **Median OS months (95% CI)*** | **Log-rank p** |
| --- | --- | --- | --- | --- | --- |
| BIRC2 (high vs low) | high | 13 | 12 | 13.0 (NA–8.0) | **0.023*** |
|  | low | 7 | 2 | NR |  |
| BIRC3 (high vs low) | high | 8 | 7 | 16.0 (NA–4.0) | 0.182 |
|  | low | 9 | 4 | NR |  |
| PD-1 (high vs low) | high | 5 | 4 | 4.0 (NA–2.0) | **0.0816** |
|  | low | 8 | 4 | 16.0 (NA–10.0) |  |

*NR = median not reached. Confidence interval fields marked 'NA' indicate not estimable from the current data and KM CI inversion

**Supplementary Table 3. Univariate association with death status (exploratory)**

| **Marker** | **N** | **High_death** | **High_alive** | **Low_death** | **Low_alive** | **OR  high vs low** | **Fisher p** |
| --- | --- | --- | --- | --- | --- | --- | --- |
| group logBIRC2 | 20 | 12 | 1 | 2 | 5 | 30 | **0.007*** |
| group log BIRC3 | 17 | 7 | 1 | 4 | 5 | 8.75 | 0.131 |
| PD1 group | 13 | 4 | 1 | 4 | 4 | 4 | 0.565 |

**Supplementary Table 4. Early death analysis (OS < 9 months)**

| **Marker** | **N** | **Early death (<9 m) in HIGH** | **Not early in HIGH** | **Early death (<9 m) in LOW** | **Not early in LOW** | **OR  high vs low** | **Fisher p** |
| --- | --- | --- | --- | --- | --- | --- | --- |
| BIRC2 group | 22 | 2 | 9 | 4 | 7 | 0.3889 | 0.635 |
| BIRC3 group | 18 | 2 | 9 | 2 | 5 | 0.5556 | 1 |
| PD-1 group | 13 | 4 | 1 | 0 | 8 | inf | **0.007*** |

**Supplementary Table 5. Exploratory univariate logistic regression (intersection set) assessing the association between BIRC2/BIRC3 expression (logRQ) and dichotomized survival endpoints. Censored observations before a given cut-off were excluded from the respective model.**

| **Endpoint** | **Predictor** | **N** | **Events (y=1)** | **OR (95% CI)** | **p** |
| --- | --- | --- | --- | --- | --- |
| OS > 12 months | BIRC2 (logRQ) | 17 | 10 | 0.01 (0.00–0.95) | **0.048*** |
| OS > 12 months | BIRC3 (logRQ) | 17 | 10 | 0.40 (0.08–2.04) | 0.268 |
| OS > 20 months | BIRC2 (logRQ) | 15 | 7 | 0.01 (0.00–1.43) | **0.069** |
| OS > 20 months | BIRC3 (logRQ) | 15 | 7 | 0.44 (0.08–2.33) | 0.336 |
| PFS > 12 months | BIRC2 (logRQ) | 15 | 10 | 0.01 (0.00–1.50) | **0.069** |
| PFS > 12 months | BIRC3 (logRQ) | 15 | 10 | 0.24 (0.04–1.67) | 0.150 |
| PFS > 20 months | BIRC2 (logRQ) | 13 | 7 | 0.01 (0.00–1.91) | **0.087** |
| PFS > 20 months | BIRC3 (logRQ) | 13 | 7 | 0.30 (0.05–1.98) | 0.213 |


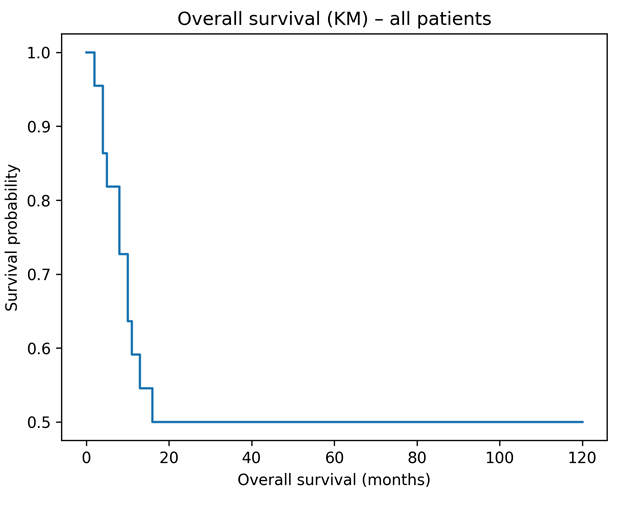


**Supplementary Figure 1. Kaplan–Meier overall survival curve for the whole cohort.**


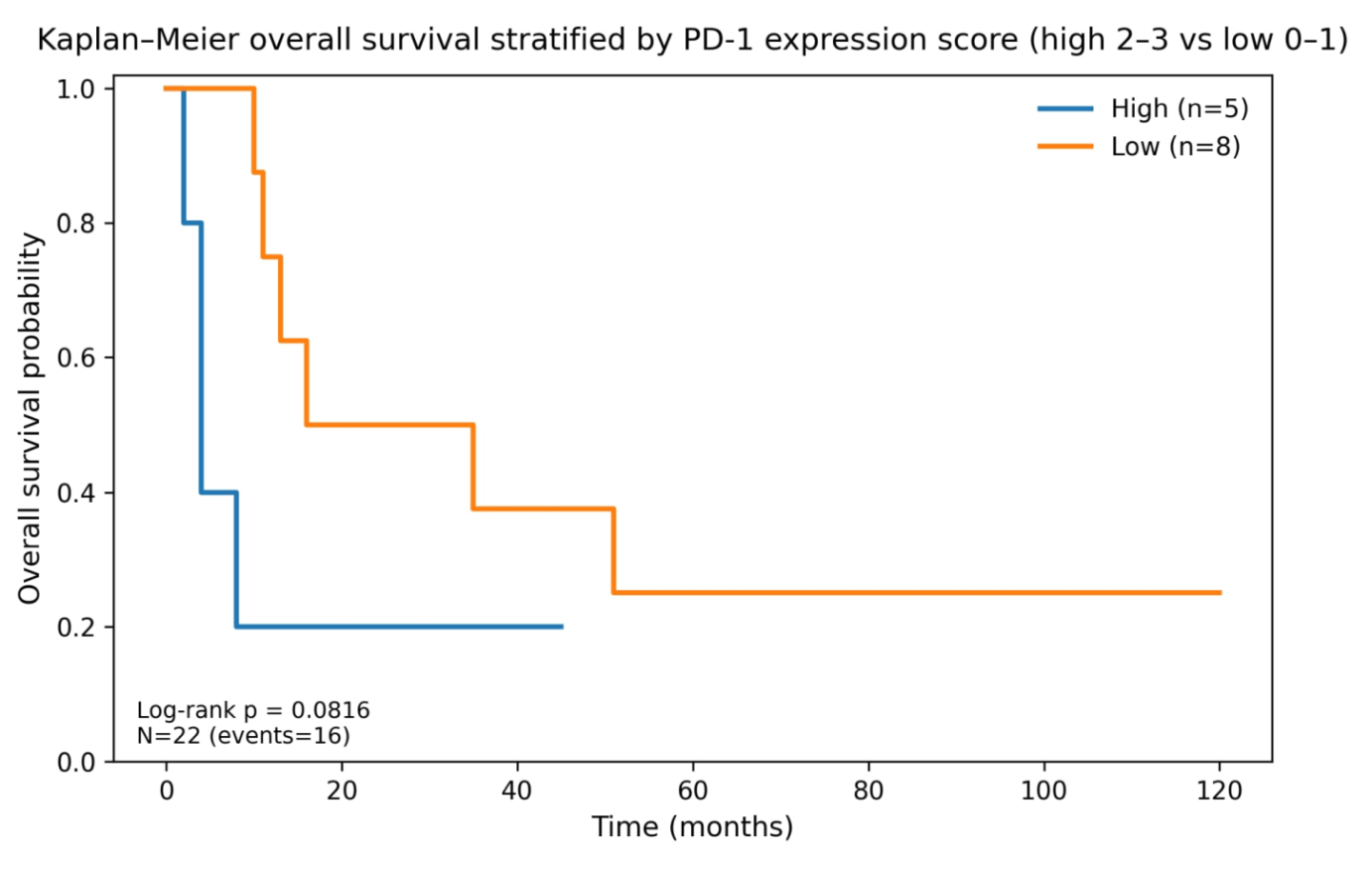


**Supplementary Figure 2. Kaplan–Meier overall survival stratified by PD-1 group (high vs low; low=0–1, high=2–3). log-rank p = 0.0816 (N=13; high=5, low=8)**


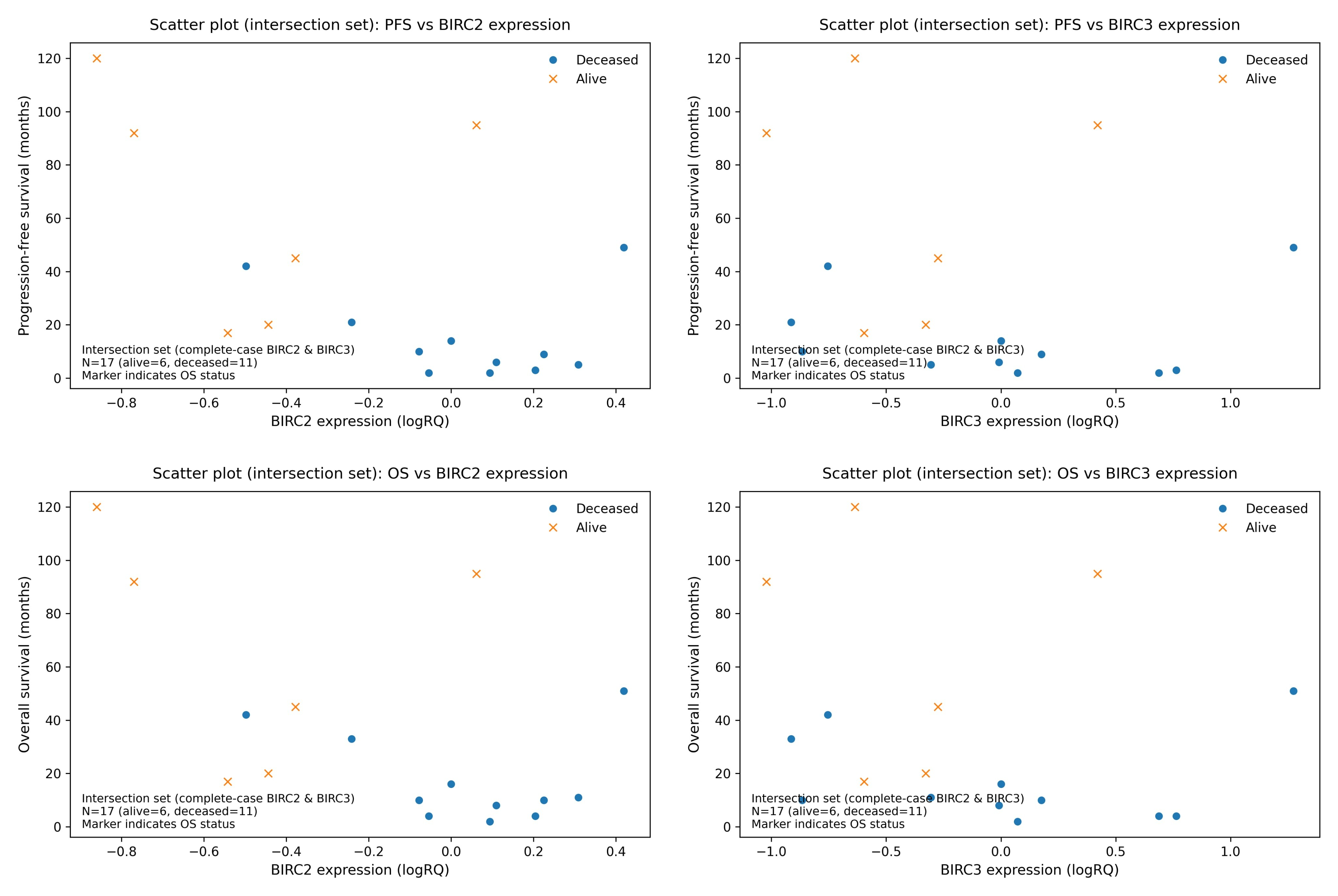


**Supplementary Figure 3. Scatter plots OS/PSF vs BIRC2/BIRC3 espression**


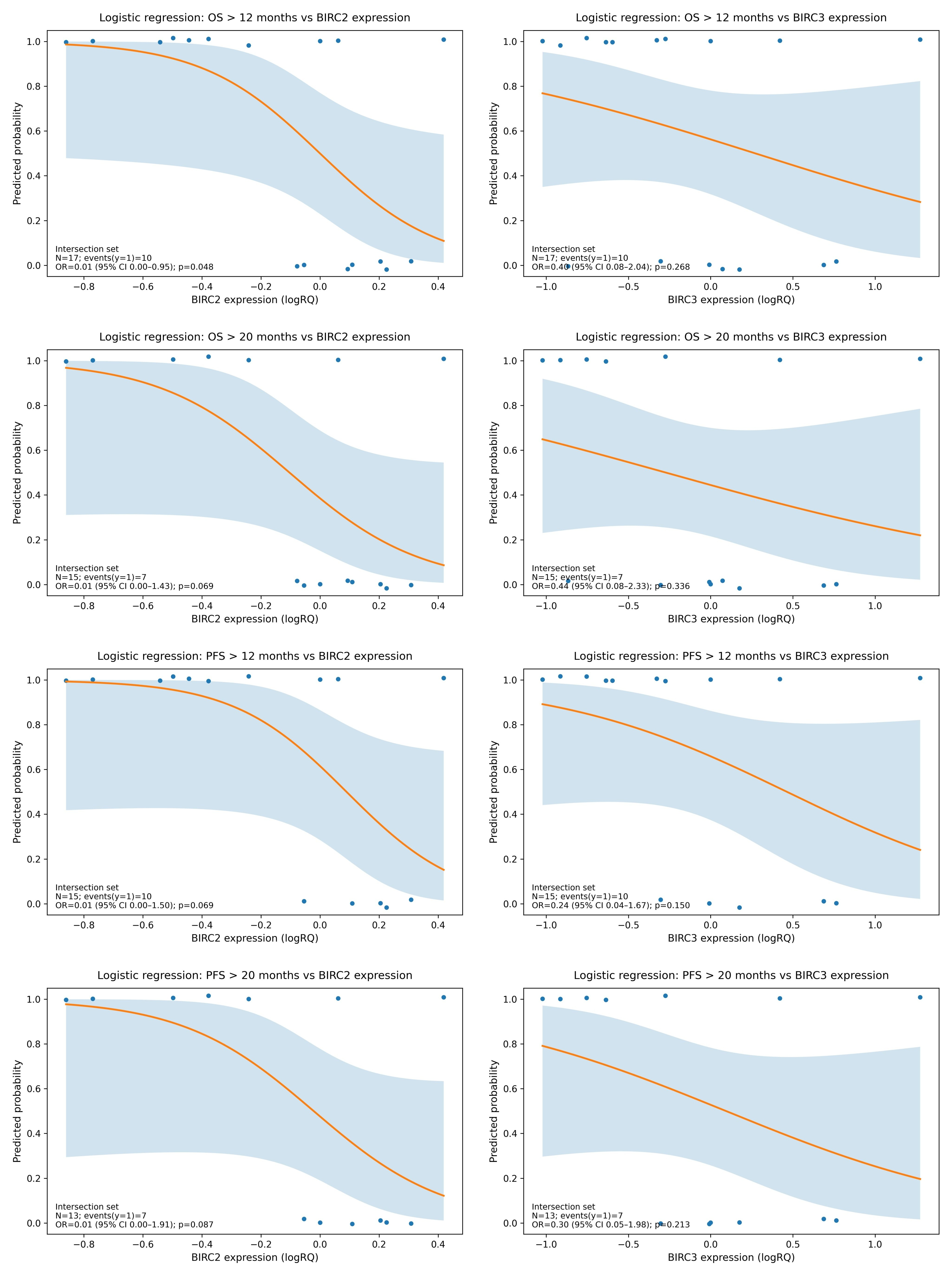


**Supplementary Figure 4. Logistic regression OS/PSF endpoints vs BIRC2/BIRC3 expression**
